# Effect of physical activity and sedentary sitting time on psychological quality of life of people with and without disabilities; A survey from Saudi Arabia

**DOI:** 10.3389/fpubh.2022.998890

**Published:** 2022-09-26

**Authors:** Aqeela Zahra, Sehar-un-Nisa Hassan, Muhammad Shehzad Hassan, Nuzhat Parveen, Jae-Hyun Park, Naveed Iqbal, Fahmida Khatoon, Mohamed Raafat Atteya

**Affiliations:** ^1^Department of Family and Community Medicine, College of Medicine, University of Ha'il, Ha'il, Saudi Arabia; ^2^College of Public Health and Health Informatics, University of Ha'il, Ha'il, Saudi Arabia; ^3^College of Medical Rehabilitation Sciences, Taibah University, Madinah, Saudi Arabia; ^4^Department of Obstetrics and Gynecology, College of Medicine, University of Ha'il, Ha'il, Saudi Arabia; ^5^Samsung Biomedical Research Institute, Sungkyunkwan University, Jangan-gu, Suwon, South Korea; ^6^Department of Obstetrics and Gynecology, College of Medicine, University of Ha'il, Ha'il, Saudi Arabia; ^7^Department of Biochemistry, College of Medicine, University of Ha'il, Ha'il, Saudi Arabia; ^8^Department of Physical Therapy, College of Applied Medical Sciences, University of Ha'il, Ha'il, Saudi Arabia

**Keywords:** psychological quality of life, disability, physical activity, sedentary sitting, Saudi Arabia

## Abstract

**Background:**

Mental and psychological health issues are on the rise globally. People with disabilities are at greater risk of poor psychological quality of life especially after covid-19 pandemic. Along with other factors physical activity (PA) may have a significant effect on mental health. This study aims to analyze the difference of PA participation and sitting time among people with and without disabilities and their association with psychological quality of life.

**Methods:**

A standard questionnaire was used to collect the data from disabled and non-disabled participants above 15 years of age. Bivariate and multivariate analysis was performed to yield statistical results.

**Results:**

Total study sample consisted of 359 participants (67.7% without disability and 32.3% with disability). Participants without disabilities reported a significantly better psychological quality of life (QOL) (Mean score = 68) as compared to the ones with disabilities (Mean score = 61), (*p* < 0.01). There was significant difference between the sitting time of two groups with longer sitting time among people with disabilities (6.1 h/day) as compared to non-disabled (5.3 h). Optimum level of PA was strongly associated with better psychological quality of life among individuals without disabilities (*p* = 0.00). Younger age (*p* = 0.00) and being single (*p* = 0.01) were significant predictors of poor psychological health among non-disables. Increase in sedentary sitting time was significantly associated with poor psychological quality of life among both groups.

**Conclusions:**

Tailored health policies to encourage PA and reduce sitting hours should be formulated to improve psychological health with special focus on individuals with disabilities. Future studies with large sample size are recommended to validate the current results and further explore the difference in association of PA and psychological wellbeing in people with and without disabilities.

## Introduction

In today's technology oriented and fast growing world there is an increase burden of mental and psychological health problems on healthcare systems (1). Psychological quality of life (QOL) further worsened by the uncertainties and lockdowns during covid-19 pandemic. Psychological QOL includes a sense of security, level of spiritual satisfaction, happiness and self-esteem. A study conducted in Saudi Arabia revealed that 23.6% of the total respondents suffered from some psychological distress or mental health problem (2). According to Saudi National Mental Health Survey, lifetime prevalence of mental disorder was 34.2% in year 2020 (3). Compared to healthy people, individuals with physical disabilities have higher prevalence of poor psychological health due to restricted mobility and social life, sedentary lifestyle, limited access to healthcare services and hostile environment (4). Considerable evidence is available to prove that individuals with physical disabilities are three times more probable to have depression than general population (4, 5).

In the past few decades there has been a growing interest of researchers and physicians in the effect of physical activity (PA) on human health. There is a strong evidence that an optimum level of PA plays an important part in maintaining the physical fitness and preventing chronic diseases like cardiovascular disease and diabetes (6, 7). In contrast, physical inactivity and sedentary sitting has been verified to double the health risks and increase the overall disease burden in both people with and without disabilities (7–9). In addition to physical benefits, previous literature also proves that regular exercise has numerous positive effects on mental and psychological health of individuals. A number of researchers determined that PA plays a significant role in reducing the symptoms of depression and anxiety and improves mood and emotional control (10–12). Furthermore, engagement in regular PA also helps in secondary prevention and onset of psychological manic episodes (10). These benefits of PA are not limited to general population but exercise has lot of progressive physical and psychological effects on people with disabilities as well (13).

In Saudi Arabia, more than one million people live with some kind of disability (14, 15). Prevalence rate of disability is 7.1% with physical disability being the most common (3.9%). This high prevalence and increasing rate of disability is a constant challenge for Saudi healthcare authorities and needs continuous research and tailored policies for improving physical and psychological QOL of people with disabilities.

Keeping in view the overall importance of psychological wellbeing, it is vital to discover the factors effecting it and its association with PA and sedentary lifestyle especially among people with disabilities. Previous studies in Saudi Arabia mostly analyzed the prevalence of mental and psychological health problems among general population (1, 2). Some studies evaluated the psychological QOL and stress level among mothers and caregivers of people with disabilities (16–18) proving high level of depression, stress and anxiety symptoms in study sample. During literature review we also found three Saudi studies which investigated the effect of PA on psychological health but these articles were also limited to individuals without disabilities (11, 12, 19). However, there is no previous study in Saudi Arabia which assessed the psychological QOL of people with disabilities and identified the factors affecting it.

Therefore, our study aims to calculate and compare the psychological QOL of people with and without disabilities using the standard World Health Organization (WHO) QOL questionnaire. We will also asses the level of PA status and time of sedentary sitting among both groups by International Physical Activity Questionnaire (IPAQ). Finally, this article will also investigate the association of PA, sitting time and demographic characteristics with psychological QOL of people with and without disabilities.

This study will explore the following research questions;

1) Is there any difference in psychological QOL of people with and without disabilities?
2) What is the level of PA and sedentary sitting time among both groups?
3) Is there any association of demographic characteristics, PA and sedentary sitting with psychological QOL?

## Materials and methods

### Study participants and ethical consideration

This study included participants with and without disability. Among disabled, only people who had physical disability were recruited. Physical disability is defined as any state of the body that creates difficulties for the person to perform daily tasks and relate with the world around them (20). All participants were aged 15 years and above comprising both males and females. Participants involved in this study were Saudi citizens currently residing in Saudi Arabia. A total of 359 people completed the survey. People without disability accounted for 67.7%, while participants with disability composed 32.3% of the total sample. Of the people with disability, 23.3% had a mild disability, 61.2% had a moderate disability, and 15.5% had a severe disability.

Data for people with disabilities was collected from Rehabilitation Hospital Medina. This hospital provides specialized rehabilitation care to people with physical disabilities caused by stroke, amputation and accidents. Face to face interviews were conducted by hospital internees who were trained to collect data through a structured questionnaire. Keeping in view the social distancing protocols due to covid-19, data for people without disabilities was collected online by creating the survey questionnaire on google. The online link was disseminated using social media platforms including WhatsApp, emails and twitter. Questionnaire was designed in Arabic language as all participants were Arabic speakers.

Detailed purpose of the study was explained, data privacy was ensured and informed consent was taken from all the participants before filling the questionnaire. The study was conducted in accordance with the Declaration of Helsinki and approved by the Institutional Review Board of the University of Ha'il, Ha'il, Saudi Arabia, dated 13 December 2021 and approved by the university letter H-2021-229.

### Data collection and study variables

#### Independent variables

Data collection for this study took place between October to December 2021. A questionnaire was designed to collect the data from participants about demographic variables including age, gender, marital status and education level. Self-reported PA status and sedentary sitting time was assessed by International Physical Activity Questionnaire Short Form (IPAQ-SF). Arabic version of IPAQ-SF has a well-known validity and reliability (21, 22). Individuals were asked that in past seven days how much time (h/min) they spent in sedentary sitting each day, when they were not performing any PA. Participants were asked about the type (mild, moderate, severe), frequency (measured in days per week), and duration (time per day) of PA in past seven days. Status of PA was analyzed by metabolic equivalent of task (MET)-min per week. MET is the energy expenditure at the time of rest. People with more vigorous PA have high MET-min as compared to inactive people. Further details on calculation of MET are available on IPAQ website (23) and in our previously published paper as well (20). World Health Organization (WHO) recommends that a healthy person should have at least 600 MET-min per week (24). We divided our participants in to two groups including people meeting WHO recommendation for PA (more than 600 MET-min/week) and people not meeting WHO recommendation (<600 MET-min/week).

#### Outcome variable

Psychological QOL was the outcome or dependent variable in this study. Psychological QOL was assessed using WHOQOL-BREF questionnaire which is a short form of WHOQOL-100. The Arabic version of the WHOQOL-BREF has strong reliability and validity (25, 26), and it has been widely used to evaluate QOL among people with and without disabilities (27–29).

This questionnaire is used to analyze the physical, psychological, social and environmental QOL of an individual with various items. However, in this study only psychological QOL domain was used. Each domain in this questionnaire has been reported distinctly in previous articles and represents a separate aspect of QOL (20). Psychological QOL was measured using the six-item questionnaire included in WHOQOL-BREF. These six items inquired about person's ability to concentrate, satisfaction with life, acceptance of body appearance, meaningfulness of life, sense of joy and frequency of negative feelings including anxiety and depression. These items were assessed on a 5-point Likert scale and specific calculation method was used to convert score items to QOL-100 with a minimum score of 6 and maximum of 100 for psychological QOL.

### Statistical analysis

Statistical analysis was conducted using the IBM SPSS software version 21.0. Descriptive analyses was performed using frequencies and percentage for categorical variables (age, gender, education, marital status, PA) and mean and standard deviation for continuous variables (sitting time, psychological QOL). Chi-square test was used to check the difference in PA status of two study groups (disable and non-disable). *T*-test was conducted to assess the mean scores of psychological QOL according to demographic variables and PA status among people with and without disabilities. A linear regression analysis was conducted separately for people with and without disabilities to assess the multivariate association of demographic variables, sitting time and PA status with psychological QOL. *P* value of less than 0.05 was considered statistically significant. A line graph was also plotted using excel to evaluate the relationship of sitting hours with psychological QOL.

## Results

### Demographic characteristics of study sample

The demographic characteristics of the respondents are shown in Table 1. Most of the participants were between 15 and 35 years of age (*p* < 0.01) in both groups with (45.7%) and without disabilities (66.3%). Our study sample dominantly included males among people with disabilities and females among individuals without disabilities. However, among people with disabilities 57.8% of the recruited participants were married in contrast to 45.3% people without disabilities (*p* < 0.05). A higher percentage of people without disabilities had college or university degree (54.7%) in comparison to people with disabilities who mostly had primary or secondary level education (53.4%), (*p* < 0.01).

**Table 1:** Demographic characteristics of the study participants.

**Variables**	**Disabled**	**Non-disabled**	**Chi-**	**P-value**
	**(*n* = 116)**	**(*n* = 243)**	**square**	
	***n* (%)**	***n* (%)**		
Age (years)				
Young (15–35)	53 (45.7)	161 (66.3)	33.18	0.000*
Middle age (36–55)	29 (25)	65 (26.7)		
Older (55+)	34 (29.3)	17 (7)		
Gender				
Male	72 (62.1)	42 (17.3)	72.67	0.000*
Female	44 (37.9)	201 (82.7)		
Education				
No education	27 (23.3)	4 (1.6)	61.59	0.000*
Primary or secondary	62 (53.4)	106 (43.6)		
College/University	27 (23.3)	133 (54.7)		
Marital status				
Unmarried	49 (42.2)	133 (54.7)	4.90	0.018*
Married	67 (57.8)	110 (45.3)		

^*^Specifies significant differences between disabled and non-disabled (*p* < 0.05).

### Comparison of PA status, sitting time and psychological QOL among people with and without disabilities

Table 2 shows the PA status, average sitting time and psychological QOL among both groups. Overall, only 46% of the participants met the WHO criteria for minimum PA level. 49.1% of the people with disabilities were performing adequate PA as compared to 44% of participants without disabilities (non-significant). There was a significant difference in sitting time per day among both groups with average time of 6.1 h (SD = 2.7) among people with disabilities and 5.3 h (SD = 2.2) in people without disabilities (*p* = 0.01). Additionally, participants without disabilities reported a significantly better psychological QOL (Mean score = 68) as compared to the ones with disabilities (Mean score = 61), (*p* < 0.01).

**Table 2:** Comparison of physical activity status, sitting time and psychological QOL among disabled and non-disabled.

**Variable**	**Total**	**Disabled**	**Non-disabled**	**Test value**	**P-value**
	**n (%)**	**n (%)**	**n (%)**	**Chi-square**	
Physical activity					
MET-min less than 600^1^	194 (54)	59 (50.9)	135 (55.6)	Chi-sq = 0.696	0.235
MET-min more than 600^2^	165 (46)	57 (49.1)	108 (44.4)		
	**Mean (SD)**	**Mean (SD)**	**Mean (SD)**	**T-test**	
Sitting time (h/day)	5.6 (2.7)	6.1 (2.2)	5.3 (2.9)	– 2.57	0.010*
Psychological QOL^3^	66 (17.6)	61.6 (13.2)	68.0 (19.1)	3.25	0.001*

^1^Not meeting WHO recommendation for physical activity.

^2^Meeting WHO recommendation for physical activity.

^3^Psychological QOL was assessed on a scale of 6 to 100.

^*^specifies significant differences between disabled and non-disabled (*p* < 0.05).

### Mean psychological QOL according to demographic variables among both groups

Mean scores of psychological QOL according to demographic characteristics and PA status are shown in Table 3. Among people without disabilities, younger participants had significantly lower QOL (mean = 65) as compared to middle age (mean = 73) and elderly (mean = 70), (*p* < 0.05). Unmarried participants without disabilities as well as overall reported poor psychological QOL (mean = 64) than married people (mean = 72) with *p* < 0.05. Participants who did not perform PA according to WHO criteria stated poor psychological QOL as compared to people who did optimum PA among both groups with and without disabilities (*p* < 0.05).

**Table 3:** Psychological QOL by demographic characteristics and physical activity level among disabled and non-disabled (Bivariate analysis).

	**Psychological QOL** ^**1**^		
	**mean (SD)**		
**Variables**	**Disabled**	**Non-disabled**	**Total**
	**(*n* = 116)**	**(*n* = 243)**	**(*n* = 359)**
Age (years)			
Young	62.9 (12.6)	65.6 (20.5)*	65.0 (18.9)
Middle age	60.9 (14.1)	73.2 (15.0)	69.4 (15.7)
Older	60.2 (13.4)	70.4 (13.9)	63.6 (14.3)
Gender			
Male	60.5 (13.9)	72.8 (17.1)	65.0 (16.2)
Female	63.4 (11.9)	67.0 (19.3)	66.3 (18.2)
Education			
No education	60.1 (14.0)	82.7 (3.5)	63.0 (15.2)
Primary or secondary	61.6 (14.0)	67.0 (20.4)	65.0 (18.4)
College/University	63.0 (10.3)	68.4 (18.1)	67.5 (17.1)
Marital status			
Unmarried	62.6 (13.0)	64.4 (21.6)*	63.9 (19.7)*
Married	60.9 (13.4)	72.4 (14.3)	68.0 (15.0)
Physical activity			
MET-min less than 600	54.6 (13.5)*	65.7 (19.2)*	62.3 (18.4)*
MET-min more than 600	68.8 (8.1)	70.8 (18.5)	70.1 (15.7)

^*^specifies significant differences between disabled and non-disabled (*p* < 0.05).

^1^Psychological QOL was assessed on a scale of 6 to 100 by WHOQOL-100 scoring.

**Figure 1:**
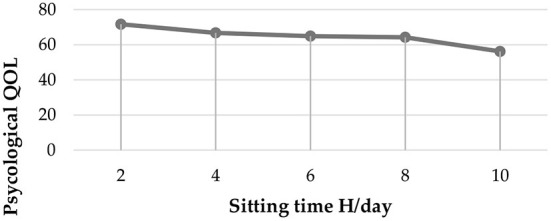
Effect of sitting time per day on psychological QOL (compute means for continuous variable).

Figure 1 shows the effect of sitting time on psychological QOL. As the average sitting time increased the psychological QOL continuously decreased as shown in the figure.

### Association of demographic variables, PA and sitting time with psychological QOL

Table 4 displays the multivariate association of demographic characteristics, PA and sitting time with psychological QOL. Middle aged people had significantly better psychological QOL as compared to young age group both in total sample (OR = 4.4, *P* < 0.05) as well as among participants without disabilities (OR = 7.5, *P* < 0.01). Married people without disabilities had 6 times more odds of having better psychological QOL as compared to unmarried individuals (*p* = 0.01). Participants with disabilities who were performing adequate PA had significantly stronger association with better psychological QOL than the ones not doing optimum PA (*P* < 0.01). Sedentary sitting time was found to be negatively associated with psychological QOL among both groups with (*P* < 0.01) and without disabilities (*P* < 0.01) as well as in total sample (*P* < 0.01). Rest of the variables did not show any significant effect on psychological QOL.

**Table 4:** Associations of demographic variables, physical activity level and sitting time with Psychological QOL (Linear Regression Analysis).

	**Outcome variable: Psychological QOL**					
	**Disabled**		**Non-disabled**		**Total**	
**Predictor variables**	**B**	***P***	**B**	***P***	**B**	***P***
Age (years)						
Young (constant)						
Middle age	– 2.06	0.50	7.58	0.00*	4.44	0.04*
Older	– 2.75	0.34	4.73	0.32	– 1.39	0.61
Gender						
Female (constant)						
Male	0.92	0.67	3.37	0.29	– 1.67	0.37
Education						
No education (constant)						
Primary or secondary	1.56	0.61	– 15.7	0.10	2.00	0.56
College/University	2.96	0.41	– 14.3	0.14	4.48	0.19
Marital status						
Unmarried (constant)						
Married	– 0.23	0.90	6.05	0.01*	3.35	0.05*
Physical activity						
MET-min less than 600 (constant)						
MET-min more than 600	11.63	0.00*	3.38	0.16	6.15	0.00*
Sitting time	– 1.44	0.00*	– 1.49	0.00*	– 1.78	0.00*

^*^indicates significant differences (*p* < 0.05); B = regression coefficient beta; *P, p*-value.

## Discussion

Up to our knowledge, this is the first study to compare PA status, average sitting time and its effect on psychological health of people with and without disabilities in Saudi Arabia. Our analysis revealed that there were no significant differences in the PA status of both groups of participants whereas people with disabilities (6.1 h) had significantly longer sitting time per day as compared to the ones without disabilities (5.3 h). These results are consistent with previous studies which also proved that majority of individuals with disabilities have sedentary behavior and longer sitting hours (30, 31). For example, a meta-analysis published in 2019 indicated that most of the people with disabilities spent more than 5.8 h sitting during wake up time which was higher than non-disabled people (4.6 h/day) (30). A previous analysis including adults with disabilities reported that individuals with mean sitting time of more than 6 h/day had 1.55 times more hazard of mortality as compared to the ones with <4 h of sitting time (32). This difference in sitting hours among both groups suggest that public health authorities should pay more attention to the sedentary lifestyle of people with disabilities and more tailored policies should be formulated to involve them in healthy activities for reducing sedentary sitting.

There was a significant difference (*p* < 0.01) in the mean scores of psychological QOL among people with (Mean score = 61) and without disabilities (Mean score = 68) with lower score in disabled. Previous studies also support our findings (3, 33). According to CDC, people with disabilities have 5 times more psychological distress in comparison to individuals without disabilities (33). However, this mean score of QOL among disabled in Saudi Arabia is poor than other developed countries like China (Mean score = 63) (34) and Portugal (Mean score = 63.8) (35). There are various factors which can affect the mental and psychological health of individuals including education level, physical health, surrounding environment and social support (36, 37). More resources should be allocated and mental health support clinics should be established especially for people with disabilities to improve their living standards.

Linear regression analysis in our study showed that among people without disabilities, middle aged group had significant association (*p* = 0.00) with better psychological QOL as compared to young participants. Mean scores of QOL were also higher is middle age participants (mean = 73) as compared to younger ones (mean = 65). Young people have specific needs for their mental and psychological comfort. Previous literature also show that mental stress and psychological problems are more common in young age (38). Most of the people in younger age group feel better with having freedom to play outside, interacting with friends and by going to school or university (39). In previous 2 years, all these activities were largely affected by lockdowns during covid-19 pandemic. Closure of schools and universities, restriction of sports and physical activity and loss of jobs increased the sitting time and use of internet and TV. All these factors may have contributed to poor psychological QOL of youngsters as compared to middle aged people. Thus, there is a greater need to ensure socialization, providing safe play and sport activities, increase job opportunities and smooth return to educational institutions to ensure psychological wellbeing of younger population.

Our analysis also implied that married people without disabilities had positive association with better psychological QOL as compared to unmarried individuals (*p* = 0.01). Marital status and presence of a partner in life are considered important factors for better financial and social support and thus good mental health. A large cohort study in Finland revealed that being single, divorced or widowed was strongly associated with more depressive symptoms and poor mental health (40). Married relationship enhancement programs, counseling that lead to a positive bond and more concentration toward psychological health of unmarried individuals can increase the QOL. Our analysis revealed that there was no significant association of age and marital status with psychological QOL of people with disabilities which infers that there may be other factors like physical health and healthcare services which have greater effect on psychological health of disabled rather than age and marriage.

Importantly, our regression analysis revealed that disabled people who performed optimum PA had a more strong positive association (*p* = 0.00) with psychological QOL as compared to the ones who did not perform the recommended amount of PA. In our sample, participants with optimum PA reported a mean psychological QOL of 68 as compared to 54 less active participants. PA has an important role in improving the psychological health, mood and muscle strength of people with disabilities (8, 41). A report of the surgeon general explained that regular exercise can decrease the symptoms of anxiety, depression and can prevent chronic diseases among disabled population (8). However, in our study sample less than half of the people (49%) with disabilities were performing regular PA. Disabled people report more environmental barriers for performing PA as compared to non-disabled in Saudi Arabia (20). Proper education campaigns about significance of exercise, improved and safe access to gyms and parks and social support by family and friends can increase the PA level and can improve psychological and physical QOL among people with disabilities.

Additionally, there was a significant negative association of sitting time with psychological QOL among both groups with (*p* = 0.00) and without disabilities (*p* = 0.00). Many researchers support the fact that increase sedentary sitting is associated with poor psychological health and increased depression and anxiety (9). During the covid-19 pandemic people spent more than average time on TV and computer screens with restricted PA. Experts recommend that moving or walking with regular intervals even while working on computers can reduce sitting time. Increase in sport activities, replacing sitting and screen time with healthy sleep and continuous walk breaks while at work can significantly decrease the negative effects on physical and mental health.

## Limitations

Our study has few limitations. Our sample included participants with physical disability only which may have limited the generalizability of results for people with disabilities. Sample size in our analysis was relatively smaller due to difficulties in collecting the data during covid-19 pandemic but it was still comparable with previous studies. Additionally, the PA level and sitting time was self-reported. People may have over reported their PA level and duration which can cause limitations in results. Future studies with larger sample size, inclusion of people with different types of disabilities and more objective record of PA by adding questions about heart rate and respiratory rate can give a more detailed analysis of factors effecting psychological health and its comparison among two groups of participants.

Despite of these limitations this study provides a valuable insight into the psychological QOL of people with and without disabilities. Additionally, it also compares the association of PA and sitting time with psychological wellbeing among people with and without disabilities which can have important health implications.

## Conclusions

People with disabilities reported poor psychological QOL as compared to individuals without disabilities. There was no significant difference in PA status of both groups however, sedentary sitting time was longer in people with disabilities. There was a strong positive association of psychological wellbeing with performing optimum PA among disabled while sitting time was negatively effecting the psychological QOL in both groups (disabled and non-disabled). These results can provide a useful direction for future research and can guide health related authorities to design specific intervention programs to reduce sitting time, encourage sports and PA and improve access of special need populations to gyms and parks for improving their mental health. This study also suggest that certain demographic variables like age and marital status can also contribute to the psychological wellbeing of individuals without disabilities while there was no association of these variables with psychological health of people with disabilities. More studies with large datasets are needed to validate these results and further investigate the factors affecting psychological QOL of life especially among people with disabilities. Mental health awareness campaigns with targeted focus on young and unmarried individuals can also improve overall QOL of non-disabled individuals.

## Data availability statement

The raw data supporting the conclusions of this article will be made available by the authors, without undue reservation.

## Ethics statement

The study was conducted in accordance with the Declaration of Helsinki and approved by the Institutional Review Board of the University of Ha'il, dated 13 December 2021 and approved by the university letter H-2021-229. Informed consent was obtained from all subjects involved in the study.

## Author contributions

Conceptualization: S-u-NH and AZ. Methodology: S-u-NH, NI, and NP. Software, investigation, writing—original draft preparation, project administration, and funding acquisition: AZ. Validation: MH, AZ, NI, and S-u-NH. Formal analysis: AZ, FK, and NP. Resources: MH. Data curation: MH and FK. Writing—review and editing: NP and J-HP. Visualization: S-u-NH and J-HP. Supervision: AZ and J-HP. All authors contributed to the article and approved the submitted version.

## Funding

This research was funded by the research deanship at University of Ha'il grant number RG-21 007.

## Conflict of interest

The authors declare that the research was conducted in the absence of any commercial or financial relationships that could be construed as a potential conflict of interest.

## Publisher's note

All claims expressed in this article are solely those of the authors and do not necessarily represent those of their affiliated organizations, or those of the publisher, the editors and the reviewers. Any product that may be evaluated in this article, or claim that may be made by its manufacturer, is not guaranteed or endorsed by the publisher.
